# Supplementary material for: Persisting roadblocks in arthropod monitoring using non-destructive metabarcoding from collection media of passive traps
Source: PeerJ. 2023 Oct 10;11:e16022. doi: 10.7717/peerj.16022 (PMC10573316; doi:10.7717/peerj.16022)
Supplement: Supplemental Information 2 [file peerj-11-16022-s002.docx]

**Persisting roadblocks in arthropod monitoring using non-destructive metabarcoding from collection media of passive traps**

Supplemental information

# Results

## *Sequencing success for collection medium treatment*

MOTUs with at least two reads (*i.e.* to remove singletons) were investigated within different sample replication combination thresholds: from additive (MOTUs present in at least 1/6 PCR replicates) to more restrictive combinations (MOTUs present in at least 2/6, 3/6 and 4/6 PCR replicates). We found reads in two out of 11 our negative controls for the most restrictive combination parameter (4/6) and in up to nine out of 11 for the additive combination (1/6). Throughout the dataset cleaning process, MOTUs found only in positive controls were removed, as for those present in negative controls and shared with samples. This filtering towards raw dataset induced between 71.3% reads drop (from 1405 MOTUs and 10,821,027 reads to 1276 MOTUs and 3,104,116 reads) for the 1/6 additive combination and 15.8% reads drop (from 210 MOTUs and 7,169,549 to 196 MOTUs and 6,037,276 reads) for the 4/6 combination parameter (Supplementary Table III). Further filtering implied the removal of non-Arthropoda MOTUs, Arthropoda MOTUs with a similarity to reference sequence below 80%, and the merging of MOTUs with identical species identification. These filtering criteria reduced the number of MOTUs from 1276 to 495 for 1/6 PCR replicates threshold, 471 to 267 for 2/6, 294 to 198 for 3/6 and 196 to 146 for 4/6 (Supplementary Table III).

# Supplemental figures

Supplementary figure 1: **MOTU and read numbers after filtering steps of Malaise trap datasets generated with different bioinformatic demultiplexing thresholds.**

Circles represent the number of MOTUs retained for various filtering and demultiplexing stringency thresholds, with circle wideness corresponding to the associated read numbers. Bioinformatic combination parameters are defined by the number of PCR replicates in which a MOTU with a minimum of two reads has to appear to be retained (*i.e.* MOTU present with two reads in at least **1/6** PCR, overlapping **2/6**, **3/6** or **4/6** PCR replicates, coloured from lighter to darker yellow, respectively). Filtering steps are described as follow : **Raw** correspond to the dataset recovered after demultiplexing; **Arthropod only** indicates a filtering based on taxonomy to retained MOTUs identified as Arthropods only; **Similarity >80%** corresponds to a filtering based on the percentage of similarity to arthropod sequences shared with the consensus from BOLD database used for taxonomic identification and keeping MOTUs sharing at least 80% similarity only; **MT filtered** corresponds to the final dataset used for Malaise traps, with a merging of MOTU and occurrence information based on an identical species identification.

Supplementary Figure 2: **Comparison of MOTU richness for different insect taxa recovered from Malaise traps using metabarcoding of collection medium (Coll. Med.) or homogenate (Hom.) with the Uni-Minibar (U-M) or Leray/Geller (L/G) primer sets.**

Boxplot of MOTU count for collection medium (yellow) or homogenate metabarcoding (blue) with Uni-Minibar primer set or from homogenate metabarcoding using mlCOIintF/jgHCO2198 primer set (grey) of the same Malaise trap samples. Black dots represent samples considered after bioinformatic processing and data curation. Significant differences adjusted with Bonferroni correction are highlighted with ‘*’ and ‘N.S.’ stands as non-significant. Studied taxa are: (**A**) non Insecta (*i.e.* Arachnida and Collembola) (Pairwise *T*-test: 1–2: *p* = 6.6^e-03^; 1–3: *p* = 7.4^e-05^; 2–3: *p* = 1); (**B**) Diptera (Wilcoxon rank sum-test: 1–2: *p* = 0.15; 1–3: *p* = 6.0^e-09^; 2–3: *p* = 8.3^e-05^); (**C**) Hymenoptera (W-test: 1–2: *p* = 1.9^e-03^; 1–3: *p* = 7.6^e-10^; 2–3: *p* = 1.2^e-05^); (**D**) Coleoptera (W-test: 1–2: *p* = 3.9^e-03^; 1–3: *p* = 6.6^e-09^; 2–3: *p* = 4.2^e-03^); (E) Lepidoptera (W-test: 1–2: *p* = 1.4^e-02^; 1–3: *p* = 1.5^e-09^; 2–3: *p* = 3.1^e-05^); (**F**) other Insecta orders grouped (W-test: 1–2: *p* = 1; 1–3: *p* = 7.7^e-08^; 2–3: *p* = 3.5^e-04^).
