# Supplementary figures and images for: Persisting roadblocks in arthropod monitoring using non-destructive metabarcoding from collection media of passive traps
Source: PeerJ. 2023 Oct 10;11:e16022. doi: 10.7717/peerj.16022 (PMC10573316; doi:10.7717/peerj.16022)

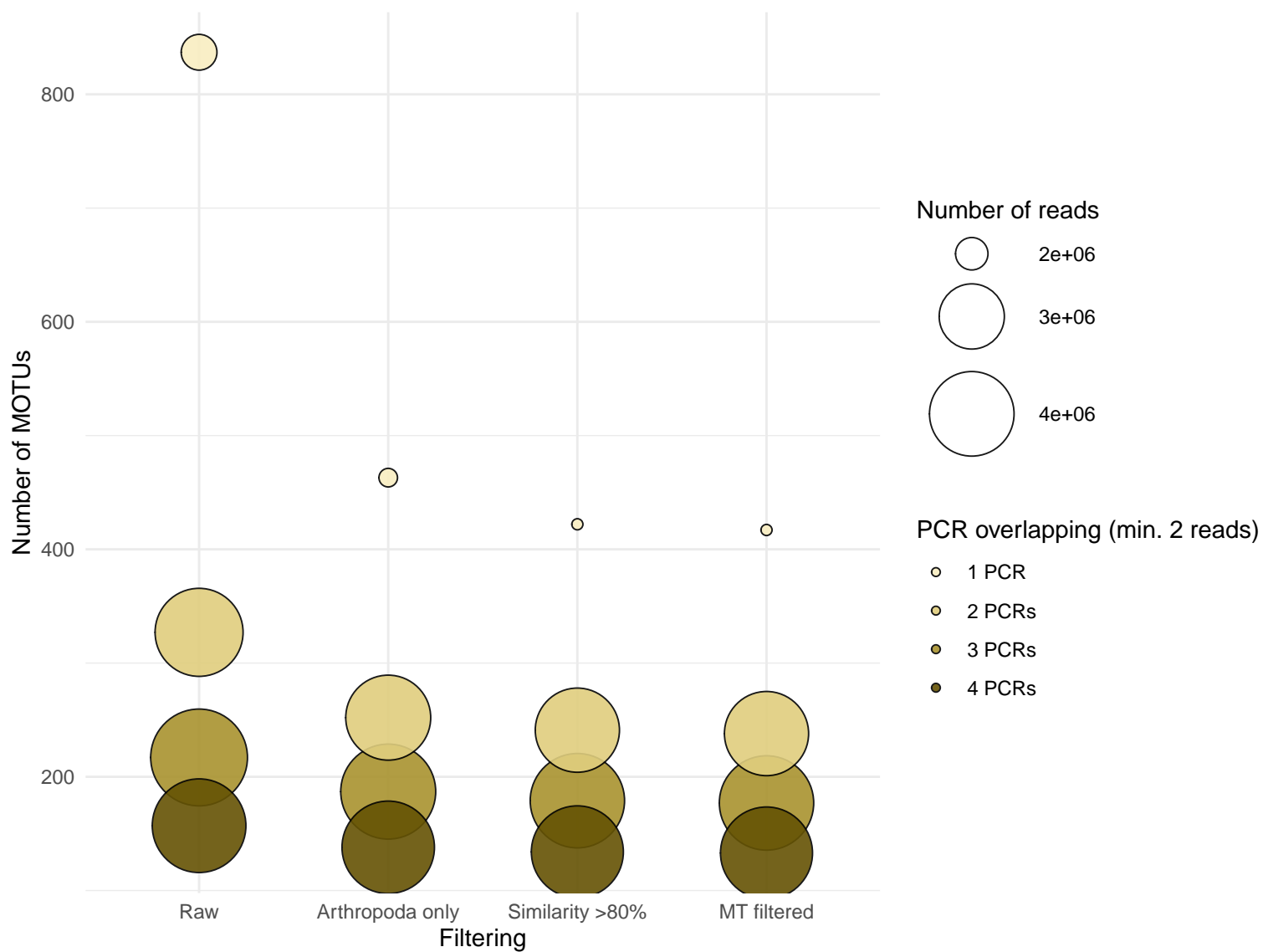

Supplement: Supplemental Information 3 — Circles represent the number of MOTUs retained for various filtering and demultiplexing stringency thresholds, with circle wideness corresponding to the associated read numbers. Bioinformatic combination parameters are defined by the number of PCR replicates in which a MOTU with a minimum of two reads has to appear to be retained (i.e., MOTU present with two reads in at least 1/6 PCR, overlapping 2/6, 3/6 or 4/6 PCR replicates, coloured from lighter to darker yellow, respectively). Filtering steps are described as follow: Raw correspond to the dataset recovered after demultiplexing; Arthropod only indicates a filtering based on taxonomy to retained MOTUs identified as Arthropods only; Similarity ¿80% corresponds to a filtering based on the percentage of similarity to arthropod sequences shared with the consensus from BOLD database used for taxonomic identification and keeping MOTUs sharing at least 80% similarity only; MT filtered corresponds to the final dataset used for Malaise traps, with a merging of MOTU and occurrence information based on an identical species identification. [file peerj-11-16022-s003.pdf]

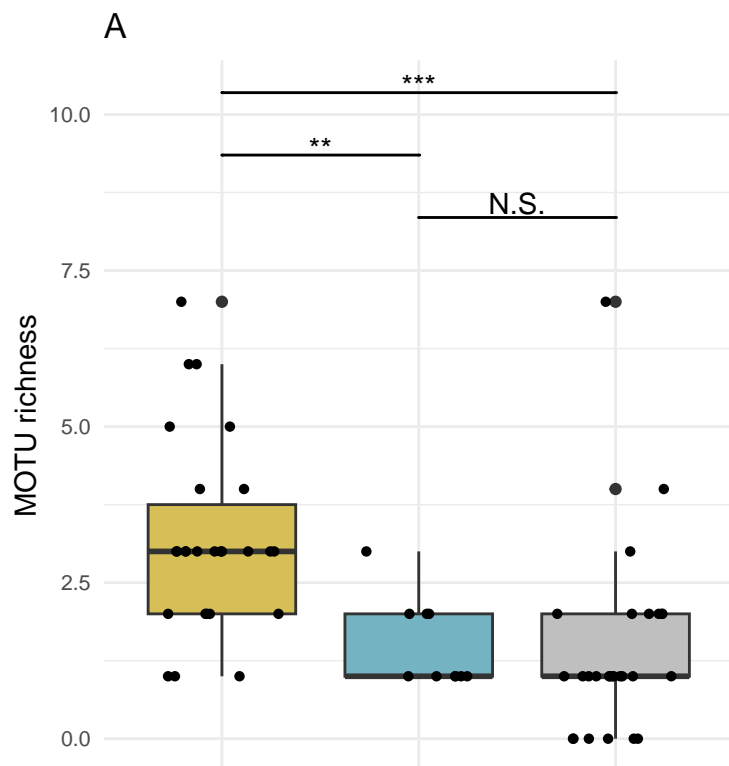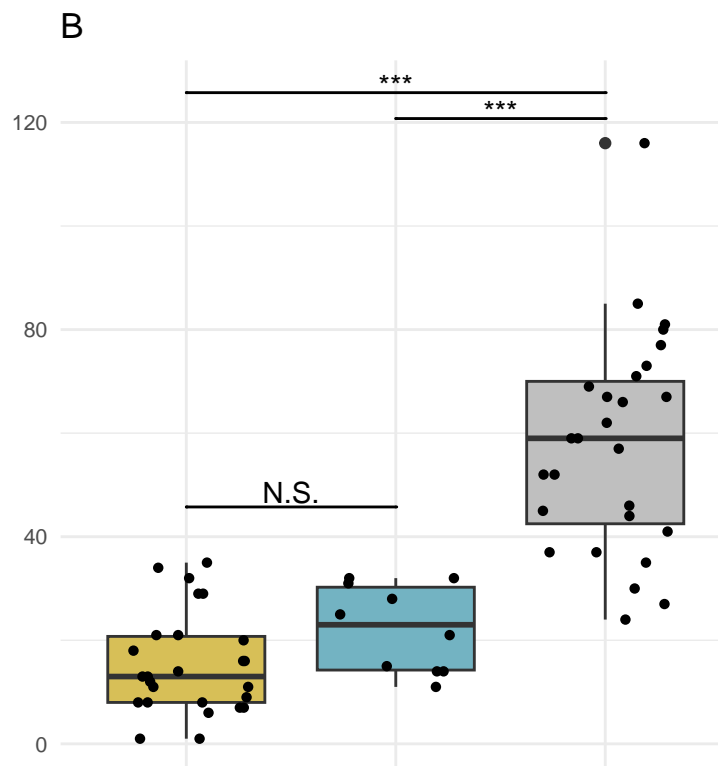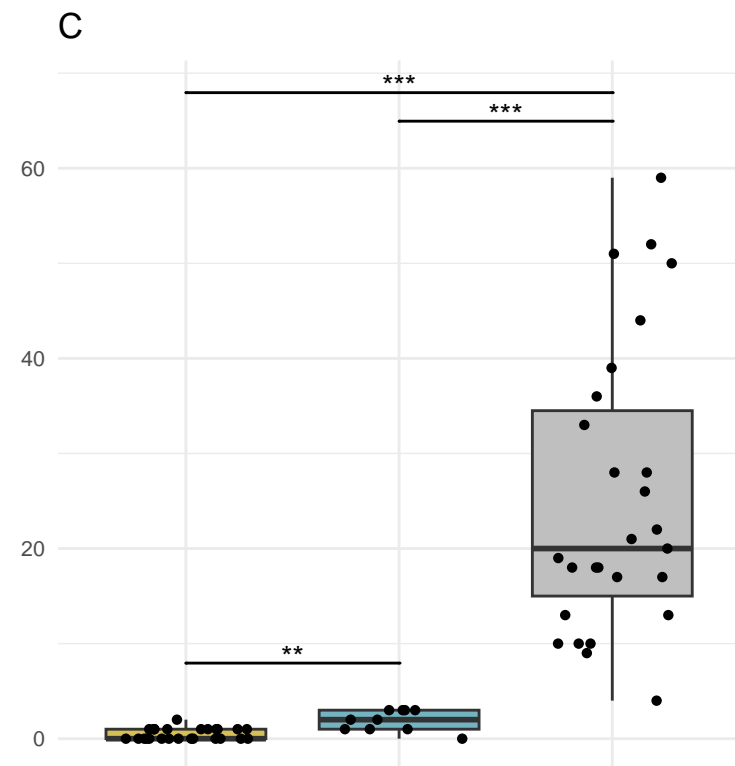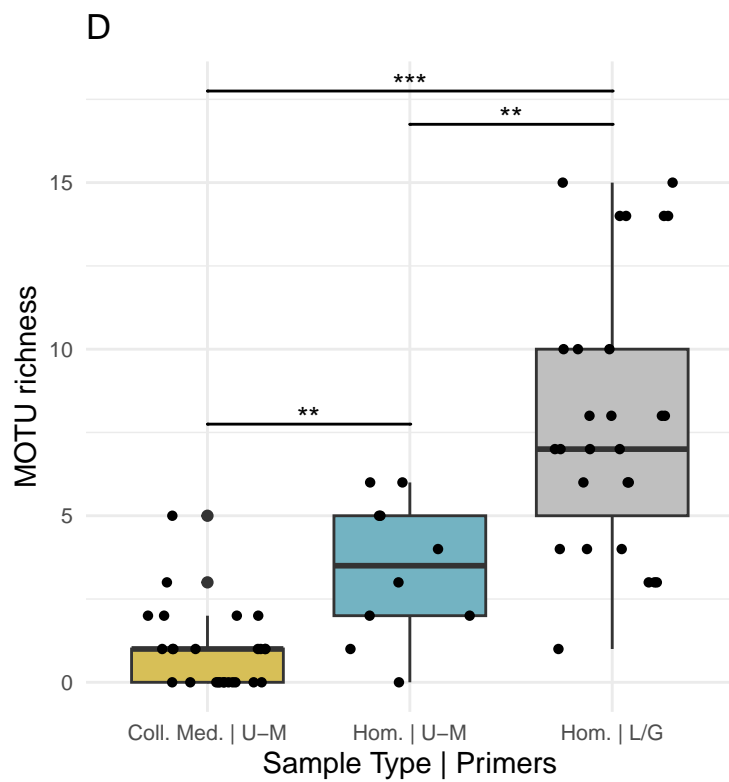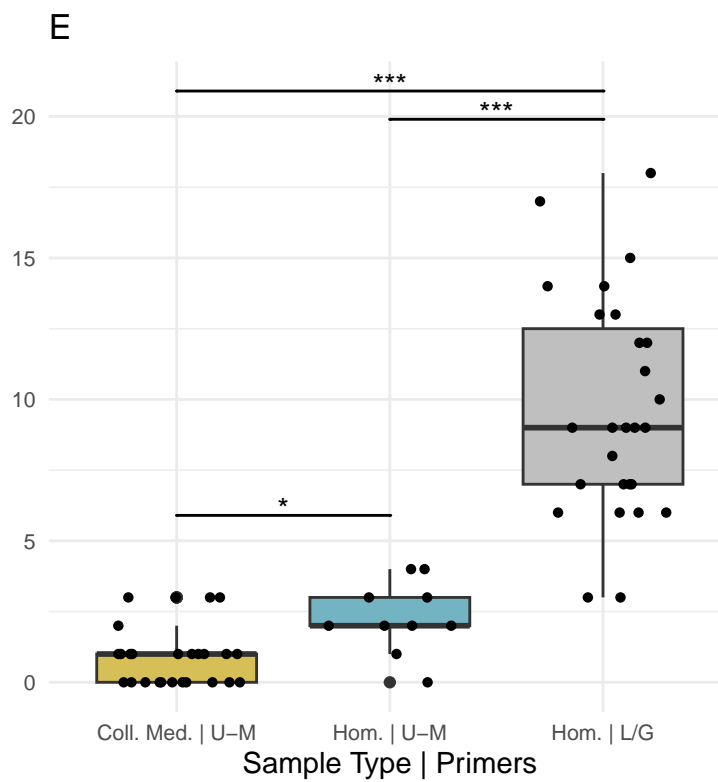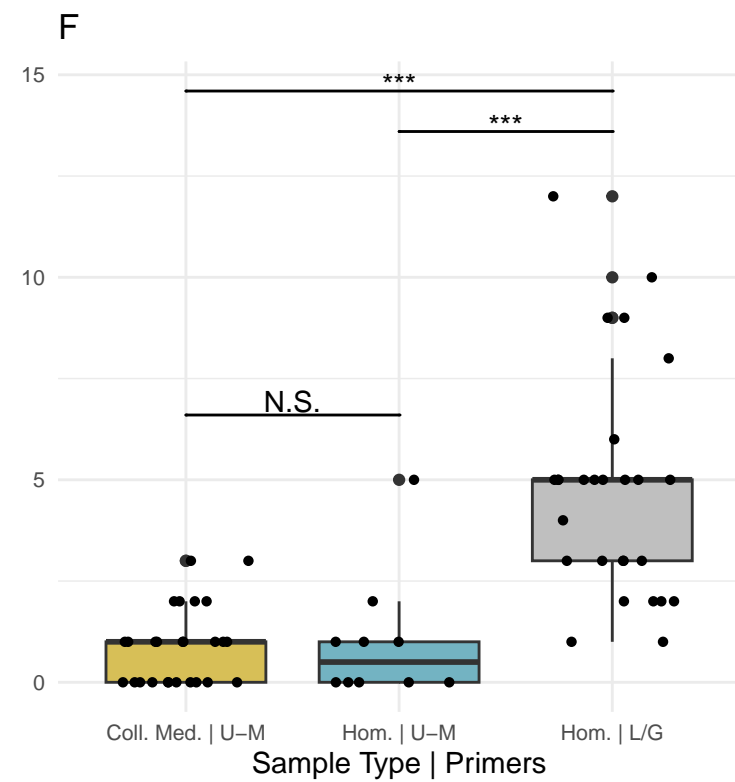

Supplement: Supplemental Information 4 — Boxplot of MOTU count for collection medium (yellow) or homogenate metabarcoding (blue) with Uni-Minibar primer set or from homogenate metabarcoding using mlCOIintF/jgHCO2198 primer set (grey) of the same Malaise trap samples. Black dots represent samples considered after bioinformatic processing and data curation. Significant differences adjusted with Bonferroni correction are highlighted with ‘*’ and ‘N.S.’ stands as non-significant. Studied taxa are: (A) non Insecta (i.e., Arachnida and Collembola) (Pairwise T-test: 1–2: p = 6.6e−03; 1–3: p = 7.4e−05; 2–3: p = 1); (B) Diptera (Wilcoxon rank sum-test: 1–2: p = 0.15; 1–3: p = 6.0e−09; 2–3: p = 8.3e−05); (C) Hymenoptera (W-test: 1–2: p = 1.9e−03; 1–3: p = 7.6e−10; 2–3: p = 1.2e−05); (D) Coleoptera (W-test: 1–2: p = 3.9e−03; 1–3: p = 6.6e−09; 2–3: p = 4.2e−03); (E) Lepidoptera (W-test: 1–2: p = 1.4e−02; 1–3: p = 1.5e−09; 2–3: p = 3.1e−05); (F) other Insecta orders grouped (W-test: 1–2: p = 1; 1–3: p = 7.7e−08; 2–3: p = 3.5e−04). [file peerj-11-16022-s004.pdf]
